# Supplementary figures and images for: FOXM1 Inhibition in Ovarian Cancer Tissue Cultures Affects Individual Treatment Susceptibility Ex Vivo
Source: Cancers (Basel). 2021 Feb 25;13(5):956. doi: 10.3390/cancers13050956 (PMC7956612; doi:10.3390/cancers13050956)

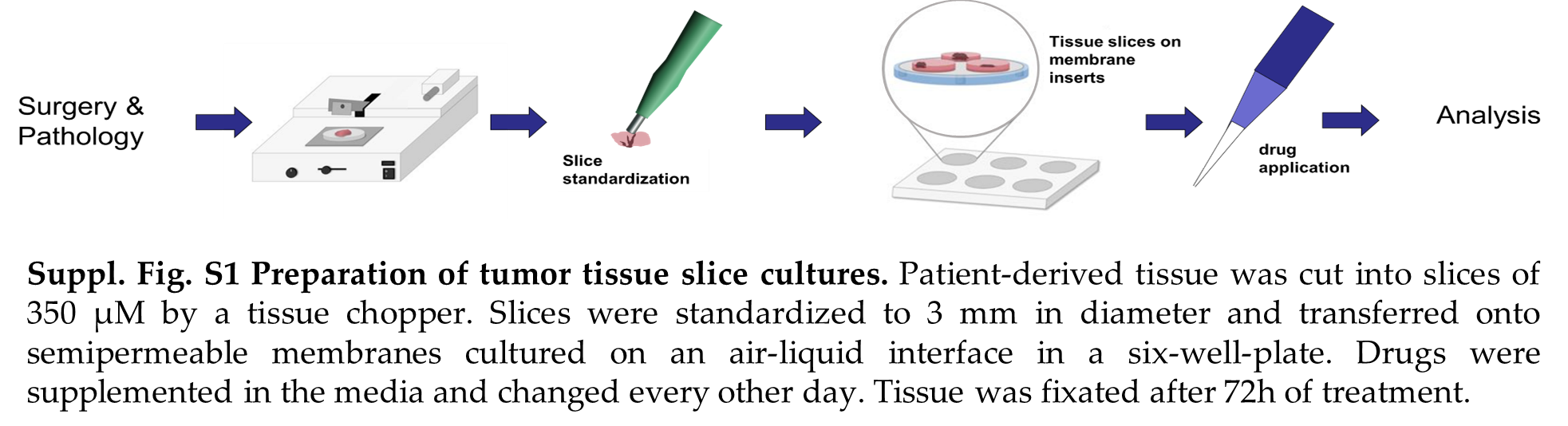

Supplement: Supplementary file 1 [file cancers-13-00956-s001.zip › Supplementary Materials/Suppl. Fig. S1.tif]

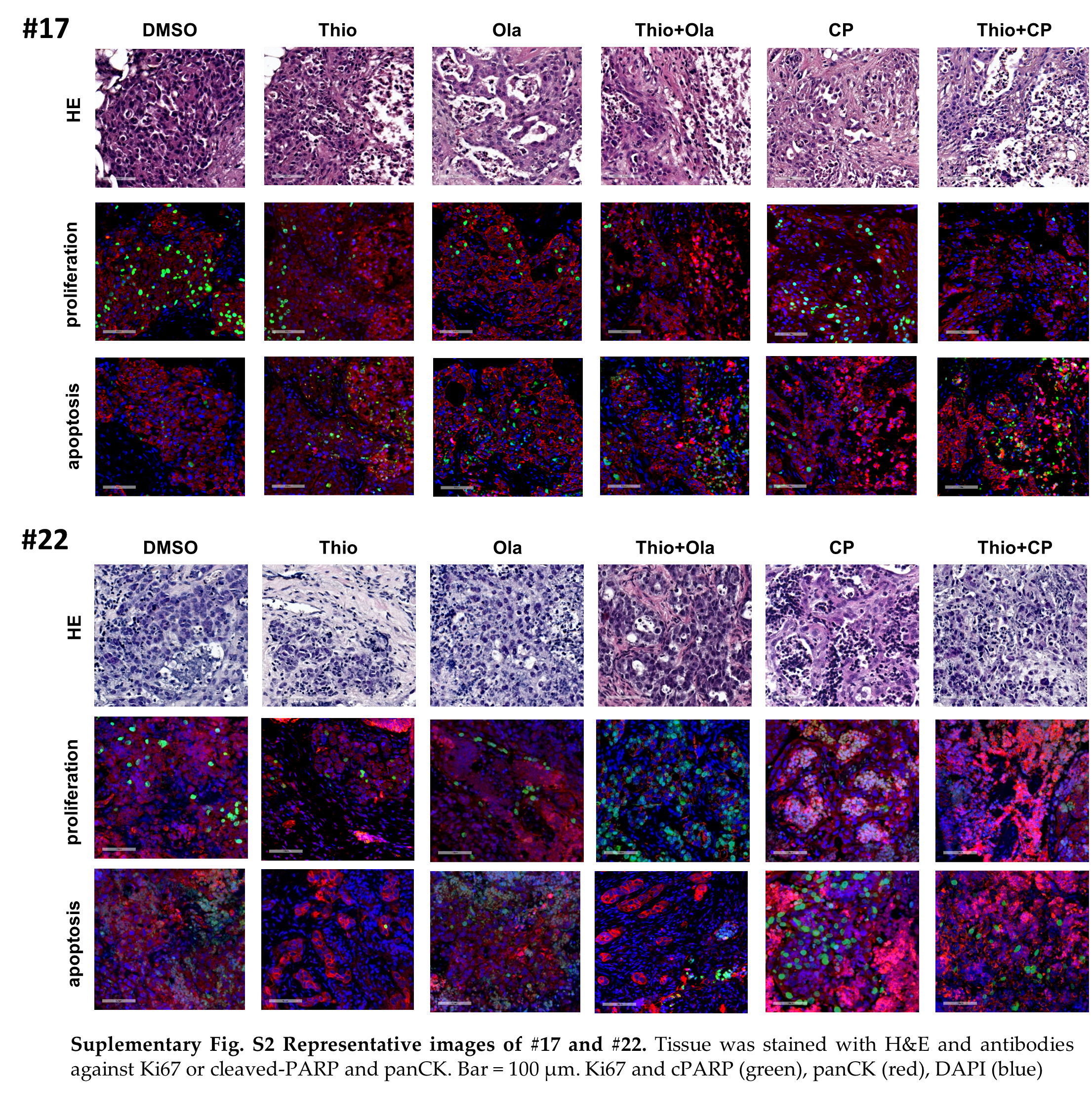

Supplement: Supplementary file 1 [file cancers-13-00956-s001.zip › Supplementary Materials/Suppl. Fig. S2.tif]

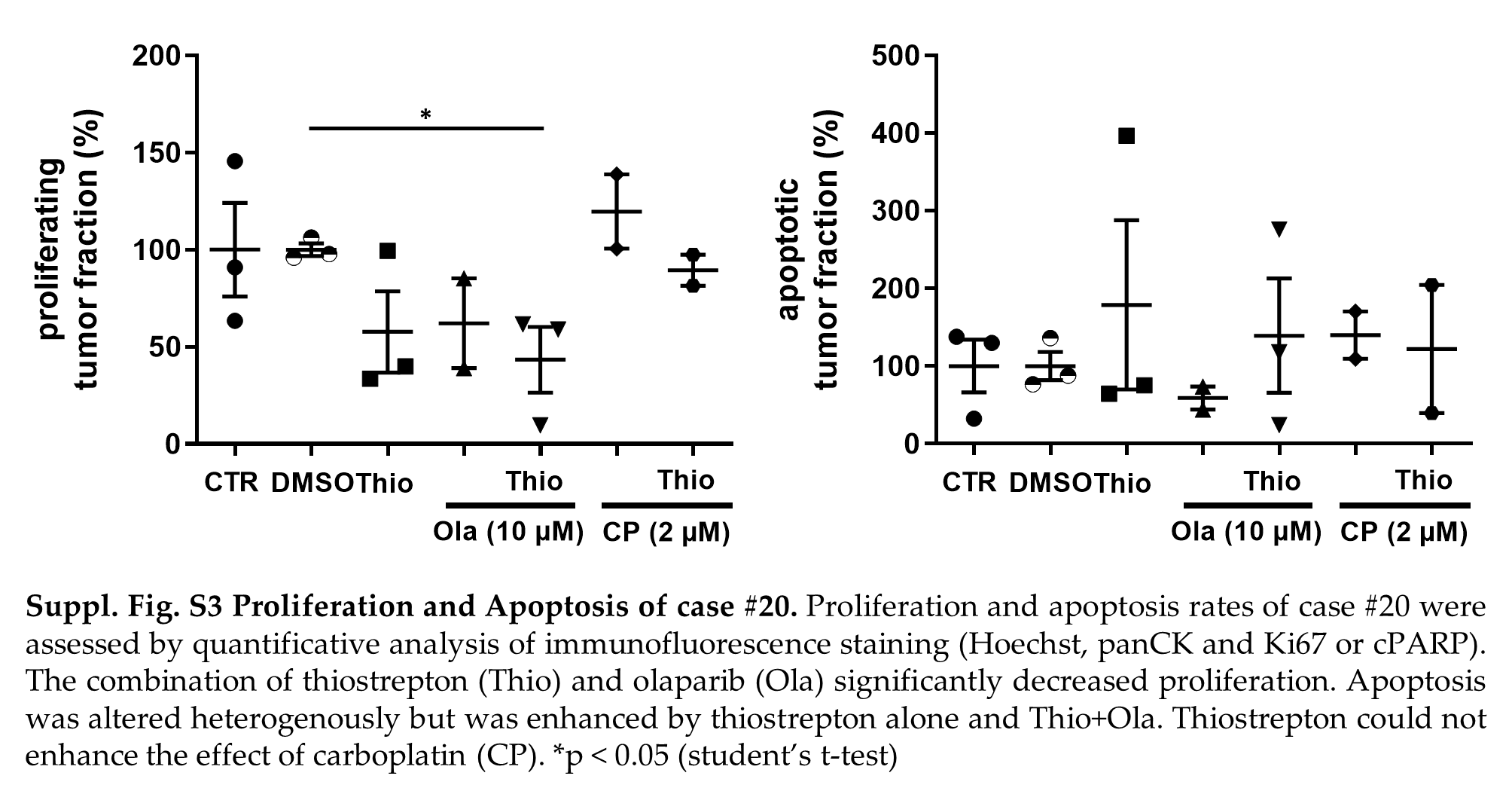

Supplement: Supplementary file 1 [file cancers-13-00956-s001.zip › Supplementary Materials/Suppl. Fig. S3.tif]

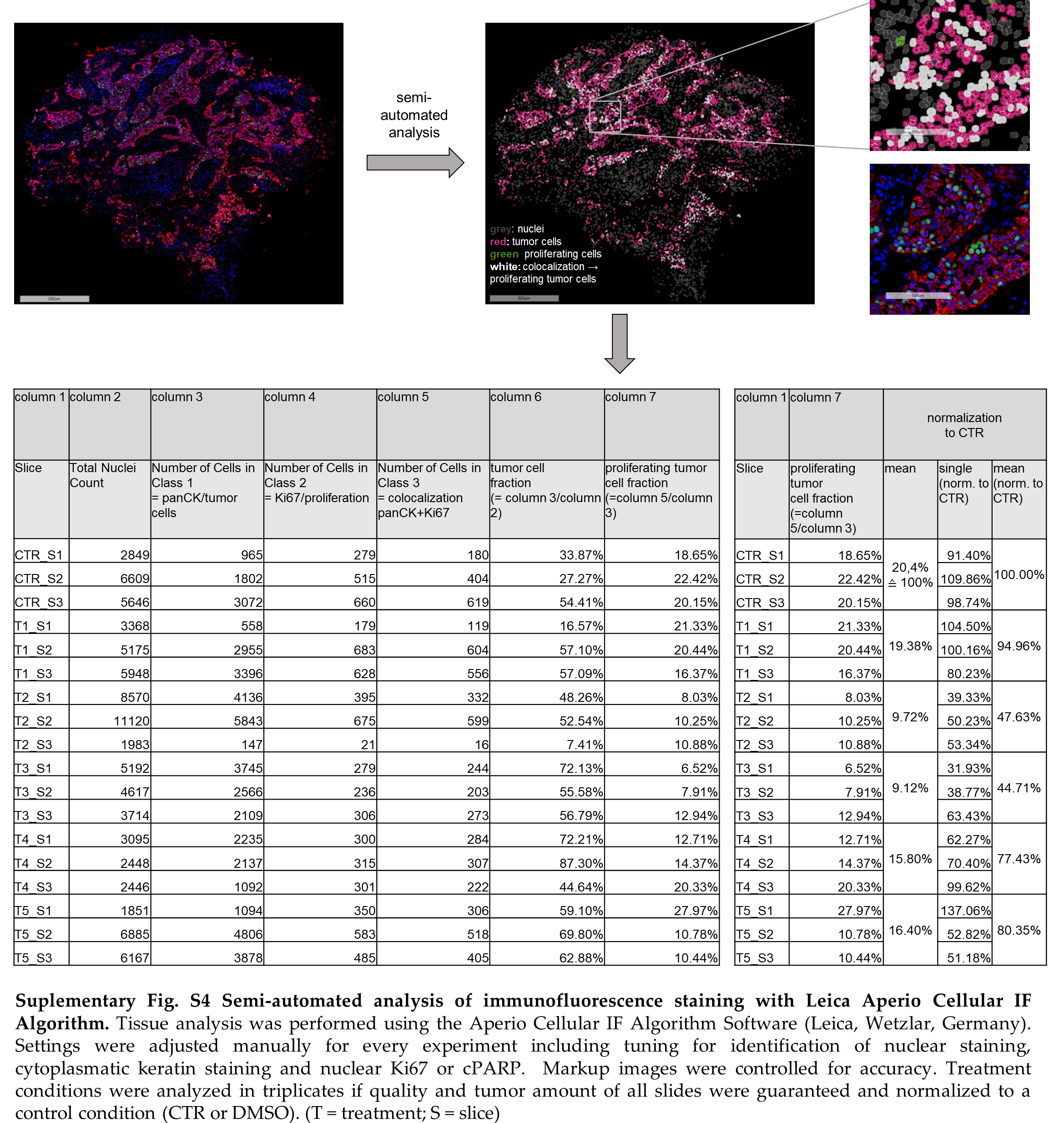

Supplement: Supplementary file 1 [file cancers-13-00956-s001.zip › Supplementary Materials/Suppl. Fig. S4.tif]

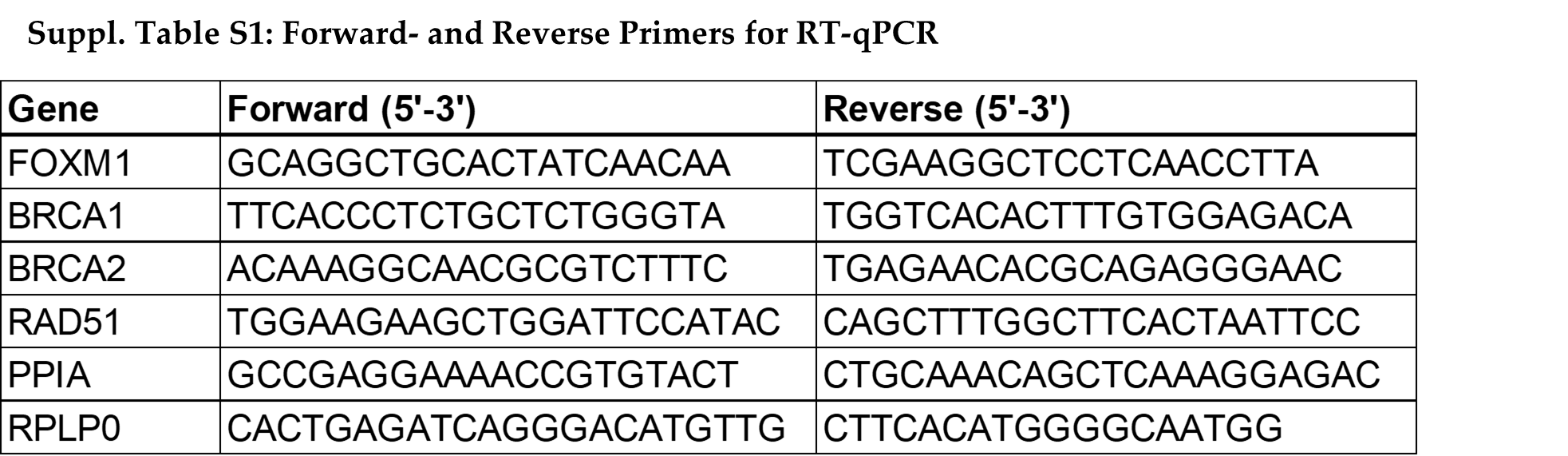

Supplement: Supplementary file 1 [file cancers-13-00956-s001.zip › Supplementary Materials/Suppl. Tbl. S1.tif]
